# Degree of Observance of the WHO Surgical Safety Checklist

**DOI:** 10.5812/traumamon.5224

**Published:** 2012-10-10

**Authors:** Abulreza Khorshidifar, Hamidreza Kadkhodaee, Zahra Zamen

**Affiliations:** 1 Department of Surgery, Tehran University of Medical Sciences, Tehran, IR Iran; 2 Department of Obstetrics and Gynecology, Tehran University of Medical Sciences, Tehran, IR Iran

**Keywords:** Surgery, Safety Checklist, World Health Organization

## Abstract

**Background:**

One of the most important goals of health care organizations is measuring and improving the quality of health care and reduction of adverse events.

**Objectives:**

The aim of this study was to evaluate the degree of observance of the WHO surgical safety checklist at two hospitals in Tehran.

**Materials and Methods:**

During this analytic cross-sectional study the degree of observance and the effects of the checklist on patient outcomes were studied. The checklist was implemented at two teaching hospitals in Tehran, Iran.

**Results:**

One hundred patients (40 ± 15 years, 44 (44%) male and 56 (56%) female) were enrolled in our study. Determination of patients diagnoses, anesthesia safety check before anesthesia, patient connection to pulse oximetry, allergic airway disturbance check and aspiration risk, confirmation of patient identity, location of surgery and surgical method by surgeon and nurse, correct numbering of materials by nurse and correct ticketing by nurse were carried out in more than 90% of cases.

**Conclusions:**

In conclusion, our study demonstrated that surgery team members comply moderately to the WHO surgery safety checklist in Iran. Iranian health care providers need to show more adherence to some items of the checklist compared to their previous routine.

## 1. Background

A recent systematic review showed that hospitals are not safe places for admitted patients, and in every 150 patients 1 patient dies as a consequence of an adverse event of medical care; two thirds of in-hospital events are associated with surgical procedures (1). It is estimated that about 234 million surgical procedures are performed worldwide per year which highlights the importance of proper in-hospital medical care for patients undergoing surgery (2).

One of the most important goals of health care organizations is measuring and improving the quality of health care and reduction of adverse events. For this reason in 2008, in an effort to improve surgical safety, the World Health Organization (WHO) released a safety checklist identifying multiple recommended practices to ensure the safety of surgical patients (3). Use of this checklist in eight hospitals showed a reduction of about 4% in major complications (4-6), and the checklist was then launched in many developed countries and most of the hospital staff use it in the operating room (7); however, the degree of observance of this checklist in developing countries such as Iran is unclear. In the present study we evaluated the degree of observance of the surgical safety checklist in two hospitals in Tehran.

## 2. Objectives

The aim of this study was evaluation of the degree of observance of the surgical safety checklist at two hospitals in Tehran.

## 3. Materials and Methods

During this analytic cross sectional study, the degree of observance and effect of the WHO surgical safety checklist on patient outcomes were studied. The checklist was released and standardized by WHO in 2008 for the first time and is applicable in all countries, although some modifications can be made when necessary. This checklist was divided into three different steps: before induction of anesthesia, before skin incision, and before the patient leaves the operating room (3).

The checklist was implemented at Firuzgar and Rasoul hospitals in Tehran, Iran from January 2009 to March 2011 in elective operations. The study included measurements of surgical procedures performed under general or locoregional anesthesia. Patients requiring interventional radiology, gastro-intestinal endoscopy, central venous catheter insertion, hospital stay less than 24 hours and surgery without anesthesia were excluded. The study was reviewed by the ethics committee of Tehran University of Medical Sciences and approved.

### 3.1. Data Collection

Data on age, sex, length of stay, number and type of surgical procedures were collected from hospital documents. All postoperative complications were recorded at the end and 19 items of the safety checklist were recorded (3).

### 3.2. Statistical Analysis

Results reported as mean ± standard deviation (SD) when normally distributed, otherwise as median and range. T-test was used for the comparison of continuous data. A P < 0.05 was defined as statistically significant. All analyses were carried out using SPSS for Windows version 16.0.

## 4. Results

At the end of the study surgical documents of 100 patients were recorded; there were 44 male (44%) and 56 female (56%) and the mean age was 40 ± 15 years. Based on the observance of the safety checklist in the operating room, patients were divided into two groups: With (A) and without observance (B). Table 1 shows clinical and baseline characteristics of the two groups of patients.

Complication rates were 14 and 18 per 100 patients in group A and B respectively. When safety checklist was scored and its three different dimensions were evaluated, in the first dimension in most patients (76%) site mark of surgery wasn't applicable before anesthesia but other items were evaluated. In the second dimension sterility of equipment was evaluated in 100 patients (100%) and in the other ones it was not. In the third dimension the lowest observance was seen in assessing equipment (which had problems) by nurses. There was no significant relationship between total score of safety checklist and age (P = 0.527) and gender (P = 0.209) of patients, but there was a significant correlation between total score and socioeconomic level and occupation of patients (P = 0.04, P = 0.026 respectively).

**Table 1:** Clinical and Baseline Characteristics of Patients With and Without Observance of WHO Surgical Safety Checklist

	Group A (with observance) N = 76	Group B (without observance) N = 24	*P*
**Age, y**	40 ± 16	41 ± 14	0.806
**Gender**			0.462
**Male**	35	9	
**Female**	41	15	
**Duration of surgery, Hours**	4.3	5.1	0.136
**Mean length of stay, Days**	8.6	8.3	0.742

## 5. Discussion

The present study assessed the compliance of Iranian surgery team members to the WHO surgical safety checklist. We evaluated adherence rate of surgery team members to each of the items of the WHO checklist separately. This allowed us to find the parts of this checklist which need more consideration in Iranian hospitals. In the study by Klei et al., only 39% of cases (in 4353 of the 11,151 patients) was the checklist fully completed by the surgical team members. In their investigation the median number of completed items was 16 (73% of the 22 items). Their results showed that after implementation of the WHO surgical checklist postoperative mortality was decreased significantly (8). The study by Haynes et al. (3) has demonstrated that using the WHO checklist decreased postoperative complication rates by 36% on average. Moreover, based on their results, the mortality decreased by a similar rate. Two other studies by deVries et al. (9) and Neily et al. (10) showed that adherence to checklists causes significant reductions in surgical mortality and morbidity. Furthermore, Weiser et al. (11) showed that use of the WHO Surgical Safety Checklist even in urgent opperations was associated with significant improvements in patient outcomes. In their study, use of the WHO checklist resulted in 36% and 62% reduction in postoperative complications and mortality rates, respectively.

“Wrong-Site Surgery” is one of the most important errorprone areas during surgery that the surgical checklist can prevent. Although the incidence of wrong site surgery is rare, it is a potentially devastating situation for both the patient and surgeon and the consequences can cause irreversible harm to the patient. A recent study revealed 5,940 cases of wrong-site surgery during 13 years (12). Based on our results, the site of surgery has been marked in only 24% of patients prior to surgery. It has been shown that after implementation of the WHO surgery checklist, some potentially lifesaving measures including an objective airway evaluation and use of pulse oximetry were also likely to be instituted, though the change in these measures was less conspicuous (13). However, our results revealed that prior to initiation of surgery in 99% and 94% of patients pulse oximetry assessment and objective airway evaluation had been performed, respectively. In 93% of our study population, antibiotic prophylaxis had been given to the patients within the last 60 minutes prior to surgery.

The WHO surgery checklist has encouraged the administration of antibiotics within the last 60 minutes prior to surgery and ideally in the operating room rather than in the preoperative wards. The checklist provides oral confirmation of appropriate antibiotic use. After implementation of the WHO checklist adherence rate to this strategy has been shown to increase from 56% to 83% and this strategy alone has been demonstrated to reduce the rate of surgical-site infection by 33% to 88% (14-20). Moreover, other studies have demonstrated that improving antibiotic delivery and timing have decreased the rates of surgical site infection by 50% or more (17, 21).

Whereas the evidence of improvement in surgical outcomes is obvious, the precise mechanism of such improvement is less clear and it seems to be multifactorial. Use of the checklist leads to changes in both systems and the behavior of the surgical team members. However, some believe that this improvement results from a mechanism known as the Hawthorne effect which is defined as “improvement in the performance due to subjects’ knowledge of being observed” (22). Besides the advantages of using the checklist in clinical practice, there are some concerns for drawbacks that merit addressing. Some health care personnel may have a skeptical attitude toward the change in their routine. Some may believe that checklist use may cause a significant increase in workload. Therefore, it should be made clear for them that the checklists only formalizes the tasks that must be performed and does not increase the workload.

In conclusion, our study demonstrated that the surgical team members have good compliance to the WHO surgical safety checklist. We revealed that Iranian health care providers need to show better adherence to some items of the checklist compared to their previous routine. However, further investigations with larger sample sizes are needed to provide complementary data and clarify the items of the checklist which need more consideration by the surgical team.
